# Alternative mating pattern in *Enoploteuthis chunii* is associated with polyandry and male-biased sex ratio

**DOI:** 10.17912/micropub.biology.001424

**Published:** 2025-01-08

**Authors:** Yuu Moriwaki, Satoshi Kusama, Anri Yamane, Md. Nur E. Alam, Noriyosi Sato, Noritaka Hirohashi

**Affiliations:** 1 Faculty of Life and Environmental Science, Shimane University, Matsue, Shimane, Japan; 2 Uozu Aquarium, Uozu, Toyama, Japan; 3 Graduate School of Natural Science and Technology, Shimane University, Matsue, Shimane, Japan; 4 Department of Fisheries, School of Marine Science and Technology, Tokai University, Shizuoka, Shizuoka, Japan; 5 Shimane University, Matsue, Shimane, Japan

## Abstract

Although cephalopods are primarily polyandrous, genetic evidence revealed rare monogamy in *Watasenia scintillans* . Here, we studied the sister species *Enoploteuthis chunii* . We found that copulation began in early July, with egg spawning occurring in early August. The sex ratio was female-biased until late August, then shifted to male-biased. After early August, the average number of sperm sacs in females exceeded that of males. As the season progresses, females began storing sperm sacs in a cryptic sperm pocket on the right lateral trunk within the mantle. This behavior is associated with male-biased sex ratio. These results suggest polyandry in *E. chunii.*

**Figure 1. Males inseminate a hidden sperm reservoir according to the increased male-biased sex ratio in Enoploteuthis chunii:**
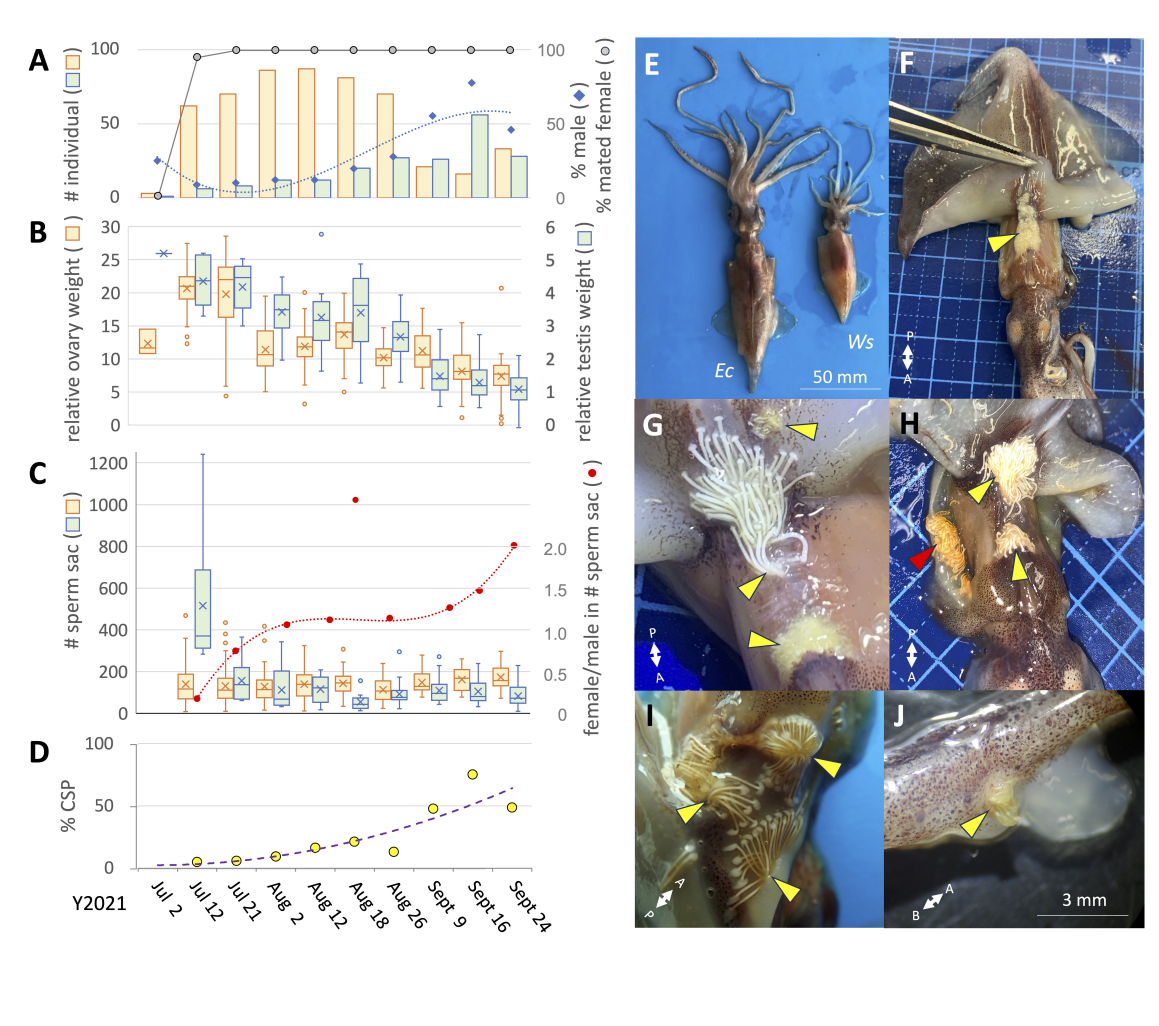
**A,** seasonal changes in the number of individuals (males in *green columns* , females in *yellow columns* ) and the sex ratios ( *a broken line* ) are depicted. The percentage of mated females in the population is also shown ( *a solid line* ). **B,** the box plots display the relative (% of body weight) ovary weight ( *yellow* ) and relative testis weight ( *green* ). **C,** the number of sperm sacs stored in males (i.e., spermatophores, *green* ) and females (i.e., spermatangia, *yellow* ) are represented in box plots. The ratios between the mean stored spermatangia and mean stored spermatophores (female/male) are plotted for each capture day ( *a broken line* ). **D,** the percentage of females with sperm sacs (spermatangia) in their cryptic sperm pocket (CSP) is shown. **E,** females of *Enoploteuthis chunii* ( *Ec, left* ) and**** *Watasenia scintillans* ( *Ws, right* ) are shown. **F-I,** representative photos show attached spermatangia ( *arrowheads* ) on females at multiple locations with different shapes, colors and sizes. *Red arrowhead* indicates a cryptic sperm pocket. **J,** spermatangia attached to the male hectocotylized (right IV) arm. Although this could happen accidentally while transferring the spermatophores, we could estimate the minimum number of spermatophores, approximately 20, to transfer at a time. Bi-directional arrows indicate the axes toward anterior ( *A* ), posterior ( *P* ) and basal ( *B* ).

## Description

The coleoid cephalopods (squids, cuttlefish and octopuses) are known to exhibit diverse mating behaviors (Hanlon & Messenger, 2018; Marian et al., 2019; Sato, 2021; Nakayama et al., 2024; Tanabe et al., 2024) . Specifically, alternative male mating behaviors have been well documented in some squid species of the family Loliginidae (Hanlon et al., 1997; Iwata et al., 2007, 2011, 2015; Apostólico & Marian, 2018; Naud et al., 2019; Hosono et al., 2024). As representative examples, males can switch their insemination sites on the female based on 1) the relative size difference between a mating pair or 2) the concurrent condition of intrasexual competition between rival males (Wada et al., 2005; Iwata et al., 2011) . Furthermore, males of *Loliolus sumatrensis* conditionally choose one or more insemination sites from three possible locations on the female body, largely based on female conditions (Azad et al., 2024) . These behavioral diversity and plasticity have been regarded as the consequences of strong sexual selective forces driven by promiscuity (Parker & Birkhead, 2013) . Therefore, female promiscuity (polyandry) is essential in creating a wide range of mating strategies and behaviors in cephalopods. However, *Watasenia scintillans* ( Fig. 1E, *right* ) presents a rare case where approximately 92% of females engage in a single copulation with a male in their lifetime (Sato et al., 2020; Alam et al., 2023) . To investigate possible reasons behind the evolution of monogamy, we examined a closely related species, *Enoploteuthis chunii* ( Fig. 1E, *left* ) *,* as both species belong to the same family, Enoploteuthidae, and share similar habitats.

Because no ecological information was available for this species, we conducted individual measurements of reproductive indices along with demographic dynamics. The specimens were obtained as bycatch of white shrimp ( *Pasiphaea japonica* ) trawling in Toyama Bay during their fishery season (from June to October) in 2021. *Enoploteuthis chunii* began to frequently appear from the second week of July 2021, with females being abundant ( Fig. 1A ). This female-biased sex ratio continued until late August, followed by a male-biased ratio until September 16, or no sex-bias on September 24 ( Fig. 1A ). No spermatangia (sperm sacs) were found in females on July 2, while over 95% of females had sperm sacs after July 12, suggesting that the onset of the massive copulation event occurs coincidentally and rapidly ( Fig. 1A, *a solid line* ). The average relative testis weight ( Fig. 1B ) and number of spermatophores (sperm sacs) males stored ( Fig. 1C ) were highest by the time of the burst copulation event (by July 12). In contrast, the average relative ovary weight (ROW) reached its peak on July 21, followed by a rapid decrease in ROW, suggesting the onset of egg spawning by females ( Fig. 1B ). Although there was no change in the number of sperm sacs stored in both males and females throughout the season (from July 12 to September 24), their ratios (female/male) showed a gradual sigmoidal increase ( Fig. 1C, *a broken line* ). Notably, the female/male ratios always exceeded 1 after August 1, suggesting that females received the sperm sacs theoretically from two or more males. Anatomical investigations indeed identified two locations that were preferentially used as attachment sites: on the dorsal trunk around the posterior neck area inside the mantle cavity and the overlaying inner surface of the dorsal mantle ( Fig. 1F ). The attached spermatangia were often seen in different sizes, shapes ( Fig. 1G, *arrowheads* ) and colors ( Fig. 1H, *arrowheads* ). Due to these differences in attached spermatangia, we assume that spermatangia were attached to a female multiple times with significant intervals ( Fig. 1G -J). These results suggest that *E. chunii* has a polyandrous mating system. Interestingly, we found a hidden sperm reservoir, coined cryptic sperm pocket (CSP), on the right lateral trunk at the inner mantle ( Fig. 1H, *red arrowhead* ). Initially, the CSP was rarely used for sperm storage, and its usage increased as the reproductive season progressed ( Fig. 1D ). There was a significant correlation between sex ratio (male/female) and the percentage of females that have sperm sacs in the CSP (Spearman’s rank correlation: R = 0.945, t=8.18, P< 0.001). We hypothesize that the use of alternative insemination sites is a result of competitive circumstances for copulation either before (male-male competition), after (insemination site competition), or both. In the first case (male-male competition), a male-biased sex ratio can directly increase the intensity of male-male competition for mating, potentially leading to alternative reproductive tactics. In some species of Loliginidae, smaller males are known to pursue sneaking copulation to deposit sperm sacs in the different sites than those inseminated by larger males (Marian et al., 2019) . In the second case (insemination site competition), due to competition for insemination sites, males must use the CSP as an alternative choice when sites are occupied with sperm sacs from other males. Based on the fact that the CSP was found only on the right side and the right IV arm is hectocotylized, we speculate that mating occurs in a male-parallel position.

## Methods

The squids, *E. chunii* , were obtained as bycatch items in white shrimp ( *Pasiphaea japonica* ) trawls at Toyama Bay off Iwase at depths between 150-300 m. Bi-weekly sampling was carried out from May to September 2021, totaling 12 days for the analysis used in this study. Additionally, specimens were collected in October and November 2021 (4 days) and August-October 2020 (5 days). The numbers of specimens obtained as bycatch items fluctuated significantly during the 6 months of white shrimp fishing. There were no or very few squids in May-June and after mid-October. Of note that all the specimens obtained on October 15, October 26, and November 2 were small, with a mantle length of 30.1±5.0 mm (n=22), suggesting the next generation was emerging. For quantitative demographic analysis, we established the following guidelines: specimen collection ended when either the total number reaches 100 (for larger quantities) or total collection time reaches 3 hours (for smaller quantities). Indexes of relative testis weight and relative ovary weight were calculated as 100 x testis weight/body weight and 100 x ovary weight/body weight, respectively. A cluster of sperm sacs attached to the female body (spermatangia) was retrieved, dissected into individual units and scored the number under the microscope. Similarly, the number of sperm sacs (spermatophores) in the male reproductive organ was also counted.
